# Genotype, but Not Climate, Affects the Resistance of Honey Bees (*Apis mellifera*) to Viral Infections and to the Mite *Varroa destructor*

**DOI:** 10.3390/vetsci9070358

**Published:** 2022-07-15

**Authors:** Ana K. Ramos-Cuellar, Alvaro De la Mora, Francisca Contreras-Escareño, Nuria Morfin, José M. Tapia-González, José O. Macías-Macías, Tatiana Petukhova, Adriana Correa-Benítez, Ernesto Guzman-Novoa

**Affiliations:** 1 Departamento de Medicina y Zootecnia de Abejas, FMVZ, UNAM, Cd. Universitaria, Mexico City 04510, Mexico; ramoscuellar92@gmail.com (A.K.R.-C.); adrianac@unam.mx (A.C.-B.); 2 School of Environmental Sciences, University of Guelph, 50 Stone Road East, Guelph, ON N1G 2W1, Canada; delamora@uoguelph.ca (A.D.l.M.); nmorfinr@uoguelph.ca (N.M.); 3 Departamento de Producción Agrícola, CUCSUR, Universidad de Guadalajara, Independencia Nal. 161, Autlan 48900, Mexico; franciscacon@cucsur.udg.mx; 4 Departamento de Ciencias de la Naturaleza, CUSUR, Universidad de Guadalajara, Enrique Arreola Silva 883, Ciudad Guzman 49000, Mexico; joset@cusur.udg.mx (J.M.T.-G.); joseoc@cusur.udg.mx (J.O.M.-M.); 5 Department of Population Medicine, University of Guelph, 50 Stone Road East, Guelph, ON N1G 2W1, Canada; tpetukho@uoguelph.ca

**Keywords:** *Apis mellifera*, *Varroa destructor*, DWV, BQCV, Africanized bees, susceptibility, resistance, genotype, climate, Mexico

## Abstract

**Simple Summary:**

The mite *Varroa destructor*, the viruses that it transmits, and the fungus *Nosema ceranae,* are among the main drivers of honey bee (*Apis mellifera*) colony losses. Honey bees are the main pollinators of flowering plants and contribute to one-third of food produced. Therefore, it is important to find out if there are honey bee strains that defend themselves better against these agents of disease. This study was conducted to determine if Africanized honey bees are more resistant than European honey bees to parasitic and viral diseases, as well as if their resistance is affected by climate. Consequently, the presence and levels of parasites and viruses was determined in 365 honey bee colonies of European or African ancestry in subtropical and temperate regions of Mexico. *Varroa* and *Nosema* were the most and least frequently found parasites (95% and 15%, respectively). Deformed wing virus (DWV) and black queen cell virus (BQCV) were the only viruses detected at frequencies of 38% and 66%, respectively. *Varroa*, DWV, and BQCV, were found at higher levels in colonies of European ancestry than in colonies of African ancestry. However, there were no effects of climate. Therefore, it is concluded that bee strain, but not climate, influences the resistance of honey bees to DWV, BQCV, and *Varroa*.

**Abstract:**

This study was conducted to analyze the effect of genotype and climate on the resistance of honey bee (*Apis mellifera*) colonies to parasitic and viral diseases. The prevalence and intensity of parasitism by *Varroa destructor*, or infection by *Nosema* spp., and four honey bee viruses were determined in 365 colonies of predominantly European or African ancestry (descendants of *A. m. scutellata*) in subtropical and temperate regions of Mexico. *Varroa destructor* was the most prevalent parasite (95%), whilst *N. ceranae* was the least prevalent parasite (15%). Deformed wing virus (DWV) and black queen cell virus (BQCV) were the only viruses detected, at frequencies of 38% and 66%, respectively. *Varroa destructor* was significantly more prevalent in colonies of European ancestry (*p* < 0.05), and the intensity of parasitism by *V. destructor* or infection by DWV and BQCV was also significantly higher in colonies of European descent than in African descent colonies (*p* < 0.01), although no genotype–parasite associations were found for *N. ceranae*. Additionally, significant and positive correlations were found between *V. destructor* and DWV levels, and the abundance of these pathogens was negatively correlated with the African ancestry of colonies (*p* < 0.01). However, there were no significant effects of environment on parasitism or infection intensity for the colonies of both genotypes. Therefore, it is concluded that the genotype of honey bee colonies, but not climate, influences their resistance to DWV, BQCV, and *V. destructor*.

## 1. Introduction

Diseases such as those caused by the mite *Varroa destructor* and the viruses that it vectors are among the most frequent biotic factors that have been associated with the massive loss of honey bee (*Apis mellifera*) colonies that have occurred worldwide in recent years [1,2,3,4,5]. *Varroa destructor* parasitism also inhibits the immune system of bees [6,7,8] and reduces honey yields of colonies [9]. Another condition that has been linked to colony losses and reduced honey bee lifespan is nosema disease [10,11,12]. Nosema disease is caused by the microsporidians *Nosema apis* and *N. ceranae*, which infect the midgut epithelial cells of bees. *Nosema ceranae*, in particular, has been found to cause higher colony infection levels than *N. apis*, and has been associated with slow spring colony buildup and reduced honey production in North America [13,14].

While it is evident that *V. destructor* and *Nosema* spp. can cause serious damage to colony health and productivity, only a few of the more than 60 viruses identified thus far in *Apis mellifera* [15] are known to cause damage to honey bees. Among these are deformed wing virus (DWV), acute bee paralysis virus (ABPV), Israeli acute paralysis virus (IAPV), Kashmir bee virus (KBV), chronic bee paralysis virus (CBPV), and black queen cell virus (BQCV) [16,17,18]. In most cases, viral infections remain latent for long periods without causing visible damage to bees and colonies, but, under certain circumstances, viruses can be influenced by stressing factors and replicate rapidly, which may result in the collapse of colonies. For example, studies have shown that viral infections in combination with *V. destructor* infestations represent a serious threat to colony health [19,20,21]. Varroa mites have been found to vector and transmit several viruses, including KBV, IAPV, ABPV, CBPV, DWV, and BQCV [19,22,23,24]. DWV can replicate in the mites, reaching high levels that are often transmitted to brood and adult bees, which results in the manifestation of clinical signs of the disease, such as damaged wings [24,25,26,27,28]. BQCV has also been occasionally associated with nosema disease and collapsed colonies [29,30], but its pathogenicity to bees is still not well understood.

The susceptibility of honey bees to pathogens is affected by several factors, including their genotypic background. For example, it is well established that *V. destructor* parasitism is not as damaging to neotropical Africanized honey bees (descendants of the African subspecies *A. m. scutellata*) as it is to European honey bee subspecies [31], in part due to natural resistance of these bees to the mite [32,33,34,35,36,37,38,39,40,41,42]. However, not much is known about whether the African ancestry of the bees provides some level of resistance to colonies against other diseases such as those caused by viruses and *Nosema* spp.

Another factor that might affect the prevalence and intensity of diseases of honey bee colonies is the climate of the location where colonies are managed. There is evidence that *V. destructor* infestation rates in South America are lower in colonies kept in tropical regions than in colonies managed in temperate regions [32], but it is not clear if these differences are due to climate or to the genotype of colonies. Moreover, not much is known about the effect of climate on the prevalence and intensity of nosema disease and viral infections in honey bee colonies.

While a considerable amount of evidence exists to support the notion that *A. m. scutellata* ancestry provides a certain degree of resistance to honey bee colonies against *V. destructor*, not many data have been generated to find out if bees of African ancestry are also more resistant to other diseases such as those caused by *Nosema* spp. and honey bee viruses, as well as about how climate affects the susceptibility of the bees to these diseases.

Understanding bee host–pathogen interactions is important to eventually develop management strategies to improve bee health. Therefore, this study was conducted to analyze the association of several diseases and the African and European ancestry of honey bee colonies in two different climates. We determined the prevalence and intensity of *V. destructor* infestations, *Nosema* spp., and viral infections of honey bee colonies of primarily European and African ancestry in subtropical and temperate climates of Mexico.

## 2. Materials and Methods

### 2.1. Samples and Locations

Samples of adult honey bees and brood were collected during early spring from 365 managed colonies that had not been requeened or treated against *V. destructor* for over a year. These colonies were located in 73 apiaries established in regions that have two different climates, subtropical and temperate, in the state of Jalisco, Mexico (19°24′ N, 101°59′ W). The municipalities in the subtropical regions are located at an average altitude of 1314.2 ± 24.1 masl with a range of 568–1699 masl and have average annual temperature and precipitation of 22.2 °C and 1117 mm, respectively, whereas those in the temperate regions are located at an average altitude of 2096.8 ± 34.2 masl with a range of 1700–2520 masl and have average annual temperature and precipitation of 14.1 °C and 801 mm, respectively [43].

The altitude of each apiary location was recorded with a GPS receiver (SporTrack Color, Magellan, Chino, CA, USA), and five colonies were randomly selected per apiary. The following samples were collected from each colony: (1) One sample of approx. 300 adult bees collected from the brood nest, placed into a 250 mL plastic jar containing 70% ethanol to detect and quantify *V. destructor*, as well as the mitotype and morphotype of the bees. (2) A 10 × 10 cm comb section containing capped brood cells to diagnose and quantify *V. destructor* in the brood. (3) A sample of approx. 100 adults collected from the entrance of the hive, placed in a 250 mL plastic jar containing 70% ethanol to diagnose and quantify *Nosema* spp. (4) Three adults collected from the brood nest of each of three of the five colonies for viral analyses. These bees were placed in sterile 2 mL microcentrifuge tubes containing RNAlater^®^ solution (Thermo Scientific; Mississauga, ON, Canada) to preserve the viral RNA. The samples were shipped to the University of Guelph (Guelph, ON Canada), where they were kept at −20 °C until viral analyses were performed.

### 2.2. Sample Analyses

The presence and intensity of *V. destructor* parasitism and *Nosema* spp. infection of the colonies was determined at the *Centro de Investigaciones en Abejas* (CIABE), University of Guadalajara, in Zapotlán, Mexico. The morphotype and mitotype of the bees as well as the detection and quantification of viruses were carried out at the Honey Bee Research Centre, University of Guelph, in Canada.

### 2.3. Prevalence and Intensity of Varroa destructor Parasitism

Adult bees and mites were separated by agitating the jars containing the bees and ethanol with a mechanical shaker (Eberbach, Ann Arbor, MI, USA) for 30 min as per De Jong et al. [44]. Mites and bees were counted to determine the percentage of mite infestation. The percentage of infested brood was derived after randomly opening 200 capped cells containing pupae and inspecting them under a stereoscopic microscope to look for mites parasitizing the brood in each comb section.

### 2.4. Prevalence and Intensity of Nosema *spp.* Infection

Spores of *Nosema* spp. were identified and quantified with a hemocytometer under a phase-contrast optic microscope (Olympus CX31, Microscope Co., Hicksville, NY, USA) as per Fries et al. [45], using the macerate of 60 bee abdomens per sample. DNA from spores of the positive samples was used for PCR identification of the *Nosema* species infecting the bees [46].

### 2.5. Viral Diagnosis

Samples were RT-PCR analyzed to detect DWV, BQCV, IAPV, and CBPV. These viruses had been previously reported to exist in Mexico [47,48,49,50]. Total RNA was extracted with TRIzol^®^ reagent (Fisher Scientific, Mississauga, ON, Canada) from three bees per sample following the manufacturer’s instructions. Complementary DNA was obtained by reverse transcription using 2 µg of RNA with the RevertAid H Minus First Strand Synthesis Kit (Fermentas, Burlington, ON, Canada) as per the manufacturer’s instructions.

The PCR reactions were performed in a Mastercycler (Eppendorf, Mississauga, ON, Canada). Each reaction contained 1.5 µL 10× PCR buffer (New England BioLabs, Pickering, ON, Canada), 0.5 µL 10 mM dNTPs (BioBasic Inc., Markham, ON, Canada), 0.2 µL 5U/µL Taq polymerase (New England BioLabs), 2 µL of cDNA, 8.8 µL ddH_2_O, and 1 µL of 10 µM forward and reverse primers of the corresponding virus. The primers and PCR conditions used to amplify DWV, BQCV, IAPV, and CBPV are shown in Supplementary Table S1. The PCR products were separated by electrophoresis on 1.1% agarose gels and stained with ethidium bromide. A 100 bp DNA ladder (BioBasic Inc., Markham, ON, Canada) was also used in each gel. The amplified bands were captured with a digital camera under a UV illuminator (BioDoc-It Imaging System, Upland, CA, USA).

Only two viruses were detected by RT-PCR (DWV and BQCV). The identity of these viruses was confirmed by sequencing PCR products (Laboratory Services, University of Guelph) and by blasting the sequences against sequences in the NCBI databases (GenBank acc. No. AJ489744.2 and AF183905.1 for DWV and BQCV, respectively). Identity was >96% in all cases.

### 2.6. Viral Quantification

DWV and BQCV, the only viruses detected by RT-PCR, were quantified by qRT-PCR. For DWV, the procedures and primers described by Di Prisco et al. [51] were used, whereas for BQCV, the quantification was performed as per Chantawannakul et al. [52].

The qRT-PCR reactions were carried out with a Bio-Rad CFX96^TM^ thermocycler (Bio-Rad Laboratories, Mississauga, ON, Canada). Each reaction was done in PCR Hard-Shell^®^ plates (Bio-Rad Laboratories), using 10 µL of PowerUp™ SYBRgreen™ (Supermix 2X) (Applied Biosystems, Foster City, CA, USA), 0.4 µL of forward and reverse primers at 200 nM concentration for DWV or at 400 nM concentration for BQCV, 6.4–7.2 µL nuclease-free H_2_O (Invitrogen, Burlington, ON, Canada), and 2 µL of cDNA or a synthetic gene fragment (gBlock^®^, Integrated DNA Technologies, Coralville, IA, USA) that contained the amplicon of DWV or BQCV, as a positive control. The sequences of the primers and the qRT-PCR conditions used are shown in Supplementary Table S1. To confirm the specificity of the target gene, a melt curve analysis was included after each qRT-PCR run.

To determine DWV or BQCV genome copies, calibration curves were generated with 300 bp gBlock^®^ synthetic fragments that were diluted with ds H_2_O to obtain a concentration of 10 ng/µL, which was used for serial dilutions from 10^9^ to 10^2^ copies. The amount of synthetic DNA was calculated as per Morfin et al. [53,54].

### 2.7. Mitotype of the Samples

To classify colonies by the mitochondrial DNA type of their bees (African or European mitotype), five worker honey bees per colony were subject to mtDNA analyses. DNA was extracted from the bees [55] to generate polymorphic fragments with restriction enzymes as per Pinto et al. [56]. First, a 485 bp fragment of the *Cytochrome b* gene was amplified with primers and PCR conditions shown in Supplementary Table S1. The PCR reactions were carried out with an Arktik thermocycler (Thermo Scientific, Missisauga, ON, Canada). Each 5 µL of reaction contained 0.5 µL 10× PCR buffer (New England BioLabs, Pickering, ON, Canada), 0.3 µL MgSO_4_ 20 nM (Bio Basic Inc., Markham, ON, Canada), 0.1 µL dNTPs 10 nM (Bio Basic Inc.), 1 µL of each primer, 10 µM, 1 µL of DNA, 0.1 µL Taq polymerase 5000 U/µL (Applied Biological Materials Inc., Richmond, BC, Canada), and 1 µL nuclease-free H_2_O (Invitrogen, Burlington, ON, Canada).

After amplification, the samples were digested with BglII restriction enzyme (Promega, Missisauga, ON, Canada) to produce RFLP markers, as per manufacturer’s instructions. The digested products were electrophoresed on a 2% agarose gel that was stained with ethidium bromide. The amplified bands were captured with photographs under a UV illuminator (Benchtop, BioDoc-ItM Imaging System, Upland, CA, USA). Two bands of 291 and 194 bp indicated that the restriction site was cut, which corresponds to European mtDNA, whereas when a single band of 485 bp was detected, the result was scored as African mtDNA.

### 2.8. Morphotype of the Samples

To classify colonies by the morphological type of their bees (European or African morphotype), the forewing length of workers from the sampled colonies was determined using the fast Africanized bee identification system (FABIS) [57]. Measurements were taken from 30 worker honey bees per colony and an average wing length was obtained for each of them. Colonies having worker bees with a mean wing length of 9.160 mm or longer were classified as European, whereas those having worker bees with a mean wing length of 8.690 mm or shorter were classified as Africanized. Colonies with worker bees that had wing length between 8.691 mm and 9.159 mm were considered undefined (probably highly hybridized) and thus were excluded from the analyses. 

### 2.9. Genotype of the Samples

To classify colonies by genotype (European or Africanized), only those colonies that had both European mitotype and morphotype were classified as European. Likewise, the colonies that had both African mitotype and morphotype were classified as Africanized. The data of these colonies were analyzed to find out if bees and colonies from the two different climates varied in prevalence for African and European ancestry, as well as in prevalence and intensity for parasitic diseases.

### 2.10. Statistical Analyses

To determine if there were differences for pathogen prevalence between colonies of the two mitotypes or morphotypes, the data on positive and negative colony parasitism or infection were analyzed with two-sample tests for equality of proportions. Data of continuous variables were analyzed with the Shapiro–Wilk normality test and the Bartlett test of homogeneity of variances. In all cases, the data did not comply with the assumptions of normality and homoscedasticity. Therefore, the data of some variables were transformed to normalize and stabilize variances, while those of other variables for which a suitable mathematical function was not found were analyzed with non-parametric statistical methods. For intensity of *V. destructor* parasitism and *N. ceranae* infection, the data were analyzed with Wilcoxon tests to compare colonies by mitotype, morphotype, or genotype. For viral infection loads, the data were first natural-log transformed and then colonies were compared by mitotype or by morphotype using Student *t* tests, whereas the genotype was analyzed with a two-way factorial ANOVA including genotype and climate as factors. For forewing length, the data were transformed with inverse transformation to the power of 8 before subjecting them to Student *t* tests to compare colonies by morphotype and mitotype. Finally, Spearman rank correlation analyses were performed for continuous data such as forewing length, parasitism intensity, and altitude of apiary location. The statistical package R 3.3.1 (Foundation for Statistical Computing, Vienna, Austria) was used for statistical analyses.

## 3. Results

### 3.1. Effect of Mitotype and Morphotype on Pathogen Prevalence 

Four diseases were detected in the studied colonies: varroosis, nosema disease, and viral infections caused by DWV and BQCV. Only one microsporidian species was detected in bees diagnosed with nosema disease (*N. ceranae*), and IAPV and CBPV were not detected. The most prevalent parasitic disease was varroosis, which was detected in 95% of the colonies, followed by BQCV infections in 66% of the colonies and DWV infections in 38% of the colonies. The disease with the lowest prevalence was nosema disease, detected in only 15% of the colonies sampled.

The prevalence of the diseases studied for the colonies classified according to their mitotype and morphotype are shown in Table 1. Varroosis was significantly more prevalent in the brood and adults of European honey bee colonies than in the brood and adults of Africanized colonies when classified by morphotype (χ^2^ = 8.6, *p* < 0.01, and χ^2^ = 3.9, *p* < 0.05, for brood and adult bees, respectively). However, no differences in pathogen prevalence were detected between colonies classified as European or Africanized, neither by morphotype nor by mitotype, for any of the other three diseases (*p* > 0.05).

### 3.2. Effect of Mitotype and Morphotype on Parasitism or Infection Intensity

**Figure 1:**
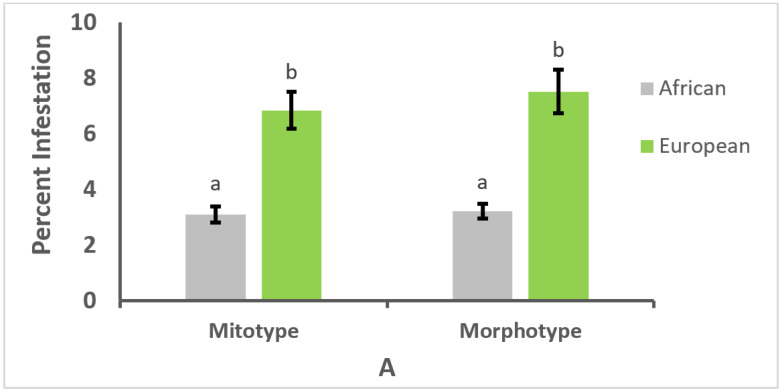

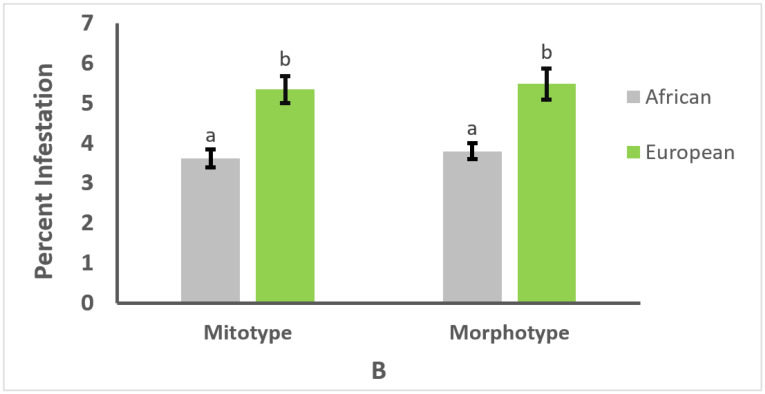
Percentage (±SE) of comb cells containing honey bee brood (**A**) or adult bees (**B**) of African or European mitotype or morphotype that were infested with *Varroa destructor* in 339 colonies. Different letters (a, b) above bars indicate significant differences between means, based on Wilcoxon tests.

Colonies of European mitotype and morphotype had significantly higher *V. destructor* infestation rates than colonies of African mitotype and morphotype, both in the brood (*W* = 9642 and *W* = 8588.5, *p* < 0.01, for mitotype and morphotype, respectively; Figure 1A) and adult bees (*W* = 10458 and *W* = 9328.5, *p* < 0.01, for mitotype and morphotype, respectively; Figure 1B).

*Nosema ceranae* infections were below injury level thresholds (161,046 ± 38,055 spores/bee), and there were no significant differences between colonies of European and African mitotype or morphotype for *N. ceranae* infection intensity (*W* = 13240 and *W* = 13,555, *p* > 0.05, for mitotype and morphotype, respectively).

**Figure 2:**
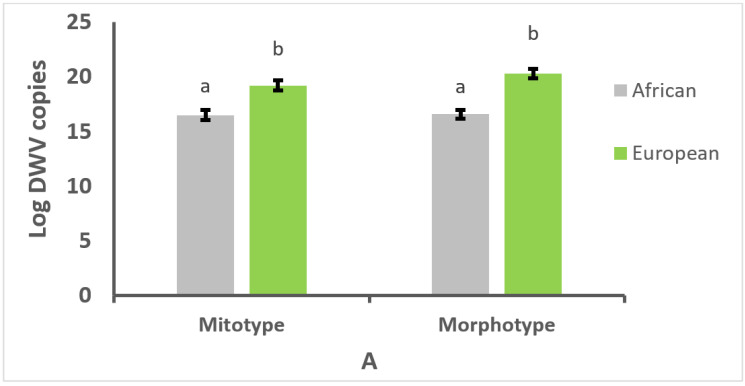

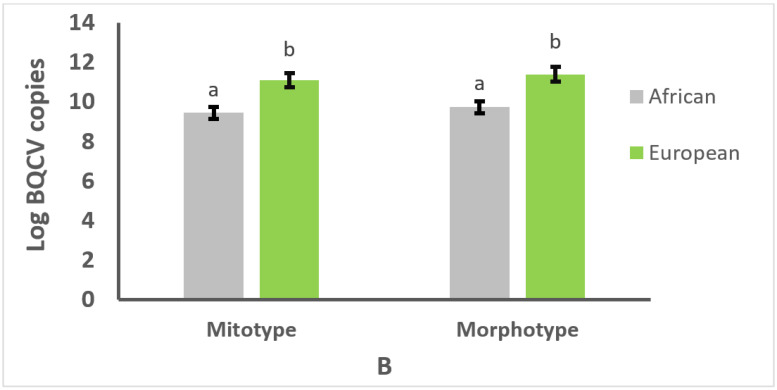
Mean number copies of deformed wing virus (DWV) (**A**) or black queen cell virus (BQCV) (**B**)/µg of RNA × 10^6^ ± SE in worker honey bees of African and European mitotype and morphotype from 76 colonies. Different letters (a, b) above the bars indicate significant differences between mitotypes and morphotypes based on Student *t* tests performed with natural logarithm-transformed data.

The infection intensity of DWV was significantly higher in colonies of European mitotype and morphotype than in colonies of African mitotype and morphotype (*t* = −4.20, df = 74, and *t* = −6.43, df = 67, *p* < 0.01, for mitotype and morphotype, respectively; Figure 2A). The number of DWV copies/µg of RNA in bees of European ancestry was on average 11 × 10^9^, which was 23 times higher than in bees of African ancestry (481 × 10^6^). Likewise, the abundance of BQCV was significantly higher in colonies of both European mitotype and morphotype compared with colonies of African mitotype and morphotype (*t* = −3.61, df = 74, and *t* = −3.51, df = 67, *p* < 0.01, for mitotype and morphotype, respectively; Figure 2B). The number of BQCV copies/µg of RNA in bees of European ancestry was on average 1 × 10^6^, which represented 5 times more viral copies than in bees of African ancestry (2 × 10^5^). Furthermore, DWV abundance was about 10,000 times higher than that of BQCV in infected bees, indicating that DWV multiplies in honey bees more than BQCV.

### 3.3. Effect of Climate on Genotype and Parasitism or Infection Intensity

**Figure 3:**
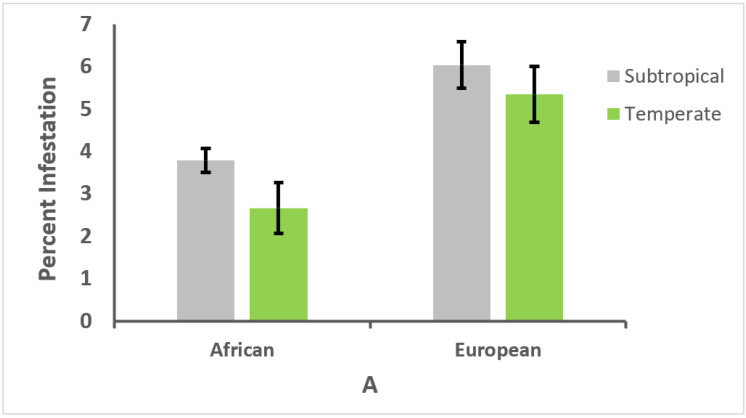

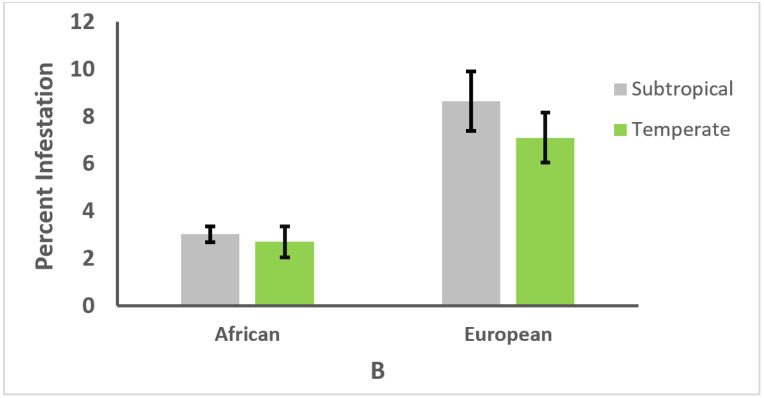
Percentage (± SE) of comb cells containing honey bee brood (**A**) or adult bees (**B**) of African or European genotype (with the same morphotype and mitotype, respectively) that were infested with *Varroa destructor* in 273 colonies located in subtropical and temperate climates.

The proportion of African and European genotypes varied significantly with climate (χ^2^ = 44.93 and χ^2^ = 14.67, *p* < 0.0001, for subtropical and temperate climates, respectively). In the subtropical climate, 66.5% of the colonies were of African genotype, and 33.5% of European genotype. Conversely, in the temperate climate, 33.3% of the colonies were of African genotype, and 66.7% of European genotype. Nevertheless, the climates did not affect the relative resistance of honey bee genotypes to the studied diseases. Colonies of European genotype had significantly higher *V. destructor* infestation rates in the brood and adult bees than colonies of African genotype in both climates (*W* = 6196 and *W* = 683, *p* < 0.01, for brood in subtropical and temperate climates, respectively; *W* = 6771 and *W* = 679, *p* < 0.01, for adult bees in subtropical and temperate climates, respectively). Furthermore, no differences in brood or adult bee infestation rates were found either for African or European genotypes when comparisons between climates were carried out for each of the genotypes (*W* = 1109 and *W* = 1651, *p* > 0.05, for brood of African and European genotypes, respectively; *W* = 1561 and *W* = 1582, *p* > 0.05, for adult bees of African and European genotypes, respectively; Figure 3A,B).

**Figure 4:**
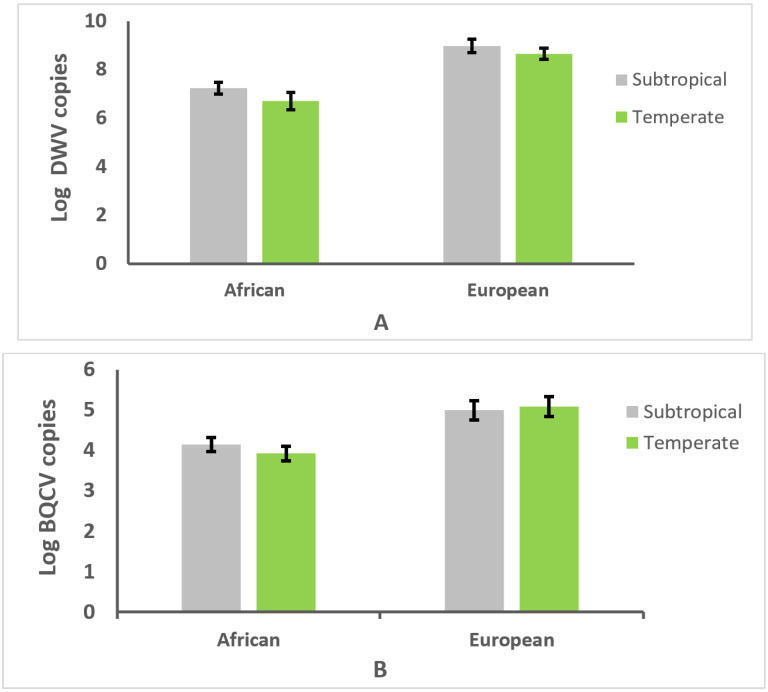
Mean number copies of deformed wing virus (DWV) (**A**) or black queen cell virus (BQCV) (**B**)/µg of RNA × 10^6^ ± SE in worker honey bees of African and European genotype (with the same morphotype and mitotype, respectively) from 59 colonies located in subtropical and temperate climates. Data transformed to natural logarithm are shown.

For DWV infection intensity, there were significant effects of genotype (*F* = 32.2, df = 1,57, *p* < 0.0001), but no effects of climate were found (*p* > 0.05; Figure 4A). Likewise, for BQCV infection intensity, there were significant effects of genotype (*F* = 17.1, df = 1,57, *p* < 0.0001), but no effects of climate were detected (*p* > 0.05; Figure 4B).

### 3.4. Relationships between Honey Bee Type, Climate, and Parasitism or Infection Intensity

The forewing length of workers from the studied colonies (morphotype) was significantly correlated with altitude, which indicates that European bee types are more frequently found in higher altitudes and temperate climates than in lower altitudes and subtropical climates. Forewing length was also correlated with *V. destructor* infestation rates in both brood and adult bees, as well as with DWV infection intensity. Additionally, *V. destructor* infestation rates in the brood and adult bees significantly correlated with DWV infection intensity (Table 2).

## 4. Discussion

This study was conducted to determine the prevalence and intensity of six diseases and the African or European ancestry of honey bee colonies in tropical and temperate climates of Mexico. Varroosis was the disease most frequently detected, whereas nosema disease was the least frequent, and the intensity of parasitism or infection varied with the pathogen. Additionally, the ancestry of the colonies was related with the altitude and climate of the location where the colonies were established. A higher proportion of colonies of European descent were located at higher altitudes in the temperate climate, whereas colonies of African descent were more frequently found at lower altitudes in the subtropical climate. Furthermore, the African and European ancestry of colonies was associated with higher and lower resistance to *V. destructor*, DWV, and BQCV, respectively, but colony genotype did not have a relationship with resistance to nosema disease.

The ancestry of honey bee colonies was partially associated with *V. destructor* prevalence. A significantly higher proportion of colonies of European morphotype were parasitized by *V. destructor* in the brood and adult bees in comparison with the brood and adults from colonies of African morphotype. These results agree with those of a study previously conducted in a region of Northern Mexico, where *V. destructor* infestations were more frequently found in colonies of European morphotype than in colonies of African morphotype [41]. However, there were no differences in mite prevalence when the ancestry of colonies was analyzed by mitotype, which might suggest that the maternal inheritance of colonies is not highly relevant for the probability of becoming parasitized. Nevertheless, this hypothesis warrants further investigation. 

Colony ancestry was also related to *V. destructor* infestation rate. Colonies of African mitotype and morphotype had significantly lower levels of parasitism than colonies of European mitotype and morphotype, both in the brood and adult bees. Moreover, worker forewing length was positively and significantly correlated with the intensity of parasitism by *V. destructor* in the brood and adult bees. Therefore, it can be inferred that at higher degree of africanization, honey bee colonies will be less infested with the mite compared to colonies with predominantly European ancestry. Medina-Flores et al. [41] also found that colonies of African genotype had lower *V. destructor* infestation rates compared to colonies of European genotype in Northern Mexico. In Colombia, where a high percentage of honey bee colonies have African mitotype (>98%), Tibatá et al. [58] reported low levels of *Varroa* infestation (<4.5%), suggesting a negative relationship between African inheritance and rates of mite infestation.

The results of this study add to the notion of Africanized bee resistance to *V. destructor* parasitism, which has been documented in a number of studies [32,34,35,38,39,59,60]. The higher relative resistance of Africanized bees to *V. destructor* is apparently due in part to a lower attractiveness of Africanized bee brood to being parasitized by the mite [34], to lower rates of reproduction of the mite in the brood of Africanized bees [40,61,62], to frequent colony swarming and evasion [63], or to greater expression of mechanisms of social immunity such as hygienic and grooming behavior, compared to bees of European descent [33,36,37,38,42,64,65,66]. It is possible that natural selection has favored the evolution of these traits to a greater extent in Africanized bee populations than in European bee populations, particularly because colonies of Africanized bees have not been subjected to chemical treatments against the mite at the intensity that European bee colonies have. Additionally, Africanized honey bee populations produce more generations at a faster rate than European honey bee populations, which would also favor a faster rate of selection of beneficial traits in tropical environments.

Nosema disease was found at a low prevalence (15%) in both types of colonies, and only *N. ceranae* was detected in the positive samples. Furthermore, no differences were found either for prevalence or intensity of *N. ceranae* infection between colonies of European and African descent. In fact, the infection levels were light (<2 × 10^5^ spores/bee) compared with reports from other regions of central Mexico, where *N. ceranae* infections of >1 × 10^6^ spores/bee have been detected [67]. It is possible that if samples are collected periodically for a long period of time, higher levels of infection and differences between bee types might be detected, which is something that warrants further investigation.

This is the first study to determine the prevalence and intensity of viral infections in honey bee colonies of European and African descent in North America. Mean DWV prevalence was 38%, whereas that for BQCV was 66%. However, no differences in viral prevalence were found between colonies of African and European ancestry. Other studies conducted in Africanized bee regions of South America have reported the prevalence of DWV and BQCV in honey bee populations, but did not relate the prevalence of the viruses with honey bee genotype. For example, in Uruguay, Antúnez et al. [68] detected BQCV in 91% of the sampled colonies and DWV in 100% of them, which indicates that these viruses are common in Uruguay. Teixeira et al. [69] found DWV and BQCV at a prevalence of 20 and 37%, respectively, in Southern Brazil, whereas in Colombia, Tibatá et al. [58] detected DWV and BQCV in 20% and 17% of the samples, respectively.

Unlike viral prevalence, the intensity of viral infections was related to the genotype of the bees. DWV and BQCV infection loads were significantly higher in bees from colonies of European mitotype and morphotype than in bees from colonies of African mitotype and morphotype. In fact, the differences in infection levels were at least 20 and 5 times higher for DWV and BQCV, respectively, in bees of European descent than in bees of African descent. Furthermore, DWV loads were about 10,000 higher than those of BQCV, indicating higher reproduction of DWV, which could have a significant impact on honey bee health. In Uruguay, Mendoza et al. [70] also found that colonies with a low degree of Africanization showed higher levels of DWV infection compared to colonies with a high degree of Africanization, which coincides with the results of this study. Additionally, in this study, the intensity of DWV infections was positively and significantly correlated with the length of workers’ forewings, suggesting that honey bees of European morphotype are more susceptible to DWV infections than those of African morphotype. Hamiduzzaman et al. [71] found evidence of possible honey bee resistance to DWV, since they observed that the virus multiplied at a slower rate over time in Africanized honey bees than in European honey bees when artificially inoculated with the virus, which supports our findings in a natural setting.

Another result of this study regarding DWV was the positive and significant correlation found between *V. destructor* infestation rates and DWV levels, which indicates that the mite plays an important role as vector and transmitter of the virus, as has been documented in European honey bees [19,26,72]. Also, other authors have supported this conclusion in Africanized bee populations. For example, Anguiano-Baez et al. [73] and Reyes-Quintana et al. [27] found that the frequency of worker bees with deformed wings, a sign of DWV infections, increased in Africanized bee colonies with higher rates of *V. destructor* infestation.

In this study, honey bees of European morphotype and mitotype were found to have higher abundance of BQCV than honey bees of African morphotype and mitotype. In Uruguay, Mendoza et al. [74] reported that although 100% of the Africanized and Italian honey bee colonies of their study were infected with BQCV, Africanized bee colonies had lower levels of BQCV infection compared with Italian bee colonies. Therefore, the results of this work and those of previous studies [70,71,74] suggest that Africanized honey bees may be more resistant than European honey bees to the replication of both DWV and BQCV.

Climate did not have a significant effect on the resistance of honey bee colonies to the parasitic and infectious agents studied. The prevalence and intensity of parasitism or infection of each of the pathogens analyzed were similar in the temperate and subtropical climates for each honey bee genotype. A couple of other studies conducted in Mexico did not find effects of climate for *V. destructor* parasitism [73,75], as in this study, and it has been found that temperature does not affect viral loads when it is moderate [76], as were the temperatures in both regions when colonies were sampled (15–25 °C). Nevertheless, the results of this and previous studies do not agree with the study by Moretto et al. [32], in which significant effects of climate were found for *V. destructor* infestation rate. Colonies in temperate climates of southern Brazil had overall higher rates of mite infestation compared to colonies kept in tropical climates, regardless of genotype. However, the study by Moretto et al. [32] was conducted for over a year and there were periods during which rates of mite infestation for a particular bee genotype were not different between environments. It is possible that if the parasitism of colonies in the two environments were analyzed for a whole season, we would also detect overall effects of climate, which warrants further investigation.

Although there is a considerable amount of evidence of a greater relative resistance of Africanized honey bees to the parasitism by *V. destructor* compared to European honey bees, more studies are needed to confirm the association between African ancestry of the bees and resistance to DWV and BQCV. Additionally, studies would be needed to discover what mechanisms might be involved in the apparent antiviral resistance of these bees, which are not necessarily the same as those that confer resistance to honey bees against *V. destructor*, since European honey bees of different genotypes that have been bred for resistance to the mite do not show differences in DWV replication [77]. Bees have mechanisms of resistance to pathogens that rely on humoral and cellular responses from the innate immune system [78]. It has been suggested that some compounds in *V. destructor* saliva (proteins or viruses) may have a suppressing effect on the innate immune system of bees [26,71]. However, Koleoglu et al. [7,8] did not find significant differences either for humoral or cellular responses between European and Africanized honey bees after 48 h post artificial inoculation with varroa mites that were infected with DWV. The researchers concluded that parasitism by *V. destructor* suppresses innate immune responses in a similar way in both European and Africanized honey bees. If this is true, it may be inferred that the greater resistance of Africanized bees to the mite and viruses may not be due to differences in the responses of their innate immune system. Nevertheless, more studies are warranted on the RNA interference (RNAi) pathways and other antiviral mechanisms that many insects, including honey bees, activate in response to viral infections [79,80], using different bee genotypes, including those of African and European descent.

The notion that *A. m. scutellata* inheritance may confer resistance to honey bee populations against *V. destructor* and viruses highlights the debate of whether some degree of Africanization could be beneficial for managed honey bee colonies. Resistance against these parasites might be an advantage to improve colony health. This is why selective breeding programs aimed at reaching equilibrium between European and African genotypes could perhaps be the best compromise to keep honey bee colonies healthy and strong in the American neotropics, for the benefit of the beekeeping industry.

Overall, the results of this study strongly suggest that the Africanization of honey bee colonies increases their resistance to *V. destructor*, DWV, and BQCV, but not to *N. ceranae*. Additionally, the climates studied did not affect the relative resistance of European or African honey bee genotypes to the above pathogens.

## Figures and Tables

**Table 1:** Prevalence of four pathogens detected in honey bee colonies of African or European mitotype (% MitA and % MitE, respectively) and of African or European morphotype (% MorA and % MorE, respectively). Data shown includes % colonies infested by *Varroa destructor* in brood (*V*. Brood) and adults (*V*. Adults), % colonies infected by *Nosema ceranae* (*N. ceranae*) and % colonies infected by deformed wing virus (DWV) and black queen cell virus (BQCV).

Pathogen	N	% MitA	% MitE	*p*	% MorA	% MorE	*p*
***V*** **. Brood**	334	76.7	84.2	0.09	75.0	88.0	0.003 *
***V*** **. Adults**	334	93.8	97.5	0.11	93.9	98.4	0.048 *
***N. ceranae***	55	13.6	18.4	0.29	16.5	15.8	0.88
**DWV**	76	30.5	45.0	0.24	32.6	48.2	0.22
**BQCV**	76	55.5	72.5	0.15	61.2	70.4	0.46

* Significance (*p* < 0.05) based on pairwise comparison tests of proportions with the Benjamini–Hochberg probability correction.

**Table 2:** Correlations of worker forewing length (W. L.) and altitude above sea level (masl) with parasitism intensity by *Varroa destructor* (*V. d.*), or infection intensity by deformed wing virus (DWV). Only significant (*p* < 0.05) correlations are shown.

Correlated Variables	N	r	*p*
**W. L.—*V. d.* brood**	339	0.32	<0.001
**W. L.—*V. d.* adults**	339	0.27	<0.001
**W. L.—DWV**	76	0.39	<0.01
**Masl—*V. d.* brood**	339	0.14	<0.01
**Masl—W. L.** ***V. d.*** **brood—DWV** ***V. d.*** **adults—DWV**	339 76 76	0.51 0.92 0.71	<0.01 <0.01 <0.05

## Data Availability

The data analyzed for the study are available from the corresponding author upon reasonable request.

## Supplementary Materials

The following supporting information can be downloaded at https://www.mdpi.com/article/10.3390/vetsci9070358/s1. Table S1: Sequences of PCR primers, PCR conditions, amplicon length, and accession numbers used in this study [22,46,47,51,52,53,54,81,82,83,84,85].
