# Supplementary material for: Genotype, but Not Climate, Affects the Resistance of Honey Bees (Apis mellifera) to Viral Infections and to the Mite Varroa destructor
Source: Vet Sci. 2022 Jul 15;9(7):358. doi: 10.3390/vetsci9070358 (PMC9320602; doi:10.3390/vetsci9070358)
Supplement: Supplementary file 1 [file vetsci-09-00358-s001.zip › vetsci-1815630-supplementary.pdf]

Table S1. Sequences of PCR primers, PCR conditions, amplicon length, and accession numbers used in this study.

Gene	Forward primer	Reverse primer	PCR conditions	Amplicon length	NCBI accession number	Reference
RpS5	5'AATTATTTGGTCGCTG GAATTG	5'TAACGTCCAGCAGAATG TGGTA	Denaturation and annealing:<br>10 cycles at 94°C 15 s, 61.8°C 30 s, 72°C 45 s; followed by 20 cycles at 94°C 15 s, 61°C 30 s, 72°C 50 s<br>Extension:72°C for 7 min	115 bp	XM_006570237.3	Hamiduzzman et al., 2010
16s rRNA *N. ceranae*	5'CGGCGACGATGTGATA TGAAAATATTAA	5'CCCGGTCATTCTCAAAC AAAAAACCG		218 bp	KX024757.1	Hamiduzzman et al., 2010
16s rRNA *N. apis*	5'GGGGGCATGTCTTTGA CGTACTATGTA	5'GGGGGGCGTTTAAAATG TGAAACAACTATG		321 bp	JQ639301.1	Hamiduzzman et al., 2010
DWV polyprotein	5'ATCAGCGCTTAGTGGA GGAA	5'CATAGATATCAGTCAAC GGAGC	Incubation: 94°C 3 min<br>Denaturation and annealing: 35 cycles at 94°C 30 s, 58°C 60 s, 72°C 60 s<br>Extension: 72°C for 10 min	642 bp	AJ489744.2	Chen et al., 2005; Guzman-Novoa et al., 2012
BQCV polyprotein	5'GGACGAAAGGAAGCCT AAAC	5'GGTGCAAGTCTCTTCCTA GT	Incubation: 94°C 3 min<br>Denaturation and annealing: 35 cycles at 94°C 30 s, 55°C 60 s, 72°C 60 s<br>Extension: 72°C for 10 min	424	AF183905.1	Tentcheva et al., 2004
IAPV	5'AGACACCAATCACGGA CCTCAC	5'GAGATTGTTTGAGAGGG GTGG	Incubation: 94°C 3 min<br>Denaturation and annealing: 35 cycles at 94°C 30 s, 55°C 60 s, 72°C 60 s<br>Extension: 72°C for 10 min	138 bp	EF219380	Maori et al., 2009
CBPV	5'CTCTAACGCCATTTGC GAGC	5'CGGTGGTCTCGTTCTTGA CA	Incubation: 94°C 3 min<br>Denaturation and annealing: 35 cycles at 94°C 30 s, 55°C 60 s, 72°C 60 s<br>Extension: 72°C for10 min	456 bp	MF175173.1	This lab
DWV helicase	5'GCGCTTAGTGGAGGAA ATGAA	5'GCACCTACGCGATGTAA ATCTG	Incubation: 95°C for 10 min<br>Denaturation and annealing: 40 cycles at 95°C 15s, 60°C 60 s<br>Extension: 68°C for 7 min	69 bp	AJ489744.2	Di Prisco et al., 2013
BQCV	5'GGTGCGGGAGATGATA TGGA	5'GCCGTCTGAGATGCATG AATAC	Incubation: 95°C for 10 min<br>Denaturation and annealing: 40 cycles at 95°C 15s, 60°C 60 s<br>Extension: 68°C for 7 min	71 bp	AF183905.1	Chantawannakul et al., 2006
Cytochrome b	5'TATGTACTACCATGAG GACAAATATC	5'ATTACACCTCCTAATTTA TTAGGAAT	Incubation: 94°C for 3 min<br>Denaturation and annealing: 30 cycles at 94°C 15 s, 50°C 15 s, 68°C 5 s<br>Extension: 72°C for 10 min	485 bp	OM203348.1	Crozier et al., 1991

References cited in Table S1

Chantawannakul, P., Ward, L., Boonham, N., Brown, M. (2006). A scientific note on the detection of honeybee viruses using real-time PCR (TaqMan) in *Varroa* mites collected from a Thai honeybee (*Apis mellifera*) apiary. *J. Invertebr. Pathol.*, 91(1), 69-73.

Chen, Y. P., Higgins, J. A., Feldlaufer, M. F. (2005). Quantitative real-time reverse transcription-PCR analysis of deformed wing virus infection in the honeybee (*Apis mellifera* L.). *Appl. Environ. Microbiol.*, 71(1), 436-441.

Crozier, Y. C., Koulianos, S., Crozier, R. H. (1991). An improved test for Africanized honeybee mitochondrial DNA. *Experientia*, *47*(9), 968-969.

Di Prisco, G., Cavaliere, V., Annoscia, D., Varricchio, P., Caprio, E., Nazzi, F., Gargiulo G., Pennacchio, F. (2013). Neonicotinoid clothianidin adversely affects insect immunity and promotes replication of a viral pathogen in honey bees. *Proc. Nat. Acad. Sci.*, 110(46), 18466-18471.

Guzman-Novoa, E., Hamiduzzaman, M. M., Espinosa-Montaño, L. G., Correa-Benítez, A., Anguiano-Baez, R., Ponce-Vázquez, R. (2012). First detection of four viruses in honey bee (*Apis mellifera*) workers with and without deformed wings and *Varroa destructor* in Mexico. *J. Apic. Res.*, 51, 342-346.

Hamiduzzaman, M. M., Guzman-Novoa, E., Goodwin, P. H. (2010). A multiplex PCR assay to diagnose and quantify *Nosema* infections in honey bees (*Apis mellifera*). *J. Invertebr. Pathol.*, *105*(2), 151-155.

Li, B., Deng, S., Yang, D., Hou, C., Diao, Q. (2017). Complete sequences of the RNA 1 and RNA 2 segments of chronic bee paralysis virus strain CBPV-BJ detected in China. *Arch. Virol.*, *162*(8), 2451-2456.

Maori, E., Paldi, N., Shafir, S., Kalev, H., Tsur, E., Glick, E., Sela, I. (2009). IAPV, a bee-affecting virus associated with Colony Collapse Disorder can be silenced by dsRNA ingestion. *Insect Mol. Biol.*, 18(1), 55-60.

Morfin, N., Gashout, H. A., Macías-Macías, J. O., De la Mora, A., Tapia-Rivera, J. C., Tapia-González, J. M., Contreras-Escareño, F., Guzman-Novoa, E. (2020a). Detection, replication and quantification of deformed wing virus-A, deformed wing virus-B, and black queen cell virus in the endemic stingless bee, *Melipona colimana*, from Jalisco, Mexico. *Int. J. Tropic. Insect Sci.*, 1-8.

Morfin, N., Given, K., Evans, M., Guzman-Novoa, E., Hunt, G. J. (2020b). Grooming behavior and gene expression of the Indiana "mite-biter" honey bee stock. *Apidologie,* 51(2), 267-275.

Tentcheva, D., Gauthier, L., Zappulla, N., Dainat, B., Cousserans, F., Colin, M. E., Bergoin, M. (2004). Prevalence and seasonal variations of six bee viruses in *Apis mellifera* L. and *Varroa destructor* mite populations in France. *Appl. Environ. Microbiol.*, *70*(12), 7185-7191.

Thompson G. J., Yockey H, Lim J., Oldroyd B. P. (2007). Experimental manipulation of ovary activation and gene expression in honey bee (*Apis mellifera*) queens and workers: testing hypotheses of reproductive regulation. *J. Exp. Zool.*, *307*A: 600-610.
